# Association Between Non-invasive Diagnostic Methods of Liver Fibrosis and Type 2 Diabetes in Pediatric Patients With Non-alcoholic Fatty Liver Disease

**DOI:** 10.3389/fped.2022.825141

**Published:** 2022-02-10

**Authors:** Aram Yang, Nayoung Jung, Sinae Kim, Ji-Eun Lee

**Affiliations:** ^1^Department of Pediatrics, Kangbuk Samsung Hospital, Sungkyunkwan University School of Medicine, Seoul, South Korea; ^2^Department of Pediatrics, Inha University Hospital, Inha University College of Medicine, Incheon, South Korea; ^3^Division of Biostatistics, Department of R&D Management, Kangbuk Samsung Hospital, Sungkyunkwan University School of Medicine, Seoul, South Korea

**Keywords:** non-alcoholic fatty liver disease, diabetes mellitus, type 2, fibrosis, child, elasticity imaging techniques

## Abstract

**Background and Purpose:**

The prevalence of non-alcoholic fatty liver disease (NAFLD) in children has been increasing associated with insulin resistance. However, there is a scarcity of related studies in children with NAFLD with type 2 diabetes mellitus (T2DM) compared to adults. We conducted this study to investigate the association between non-invasive diagnostic methods of liver fibrosis and T2DM in pediatric patients with NAFLD.

**Methods:**

We enrolled a total of 152 patients aged <18 years with NAFLD, and compared their data according to the presence of T2DM. We evaluated fibrosis by transient elastography (TE, FibroScan®), and calculated the following fibrosis scores for each patient: NAFLD fibrosis score (NFS), AST: platelet ratio index (APRI), Fibrosis-4 (FIB-4) index, and pediatric NAFLD fibrosis index (PNFI).

**Results:**

In the NAFLD–T2DM group, the NFS and mean controlled attenuation parameter in FibroScan were significantly higher than those in the nondiabetic group. The receiver operating characteristic (ROC) curve values for predicting the presence of T2DM were 0.78 for NFS, 0.64 for FIB-4, 0.62 for PNFI, and 0.61 for APRI. The cutoff HbA1c levels for predicting fibrosis progression in APRI, NFS, and PNFI were 5.7% [area under the curve (AUC) 0.74], 6.4% (AUC 0.71), and 6.4% (AUC 0.55), respectively. In the multivariate analysis, hepatosteatosis on abdomen sonography, NFS, FibroScan F, and APRI were independently associated with T2DM risk.

**Conclusions:**

We significantly characterized non-invasive fibrosis markers and elastography in pediatric NAFLD with T2DM compared with the nondiabetic group. We suggest evaluating the progression of fibrosis in the prediabetic stage in children using a combination of these non-invasive methods.

## Introduction

With the increasing prevalence of childhood obesity, non-alcoholic fatty liver disease (NAFLD) has become the most frequent cause of chronic liver disease in children and adolescents (1). Anderson et al. (2) reported that the global prevalence of NAFLD in children and adolescents was 7.6% in the general population and 34% in obese children and adolescents. NAFLD includes a broad range of disease severity, ranging from the mildest form of isolated steatosis to non-alcoholic steatohepatitis (NASH) with advanced fibrosis and cirrhosis (3). Although most patients with NAFLD do not progress to advanced fibrosis or cirrhosis, NAFLD can develop chronic liver disease that necessitates liver transplantation even in children (4). NAFLD, the hepatic manifestation of metabolic syndrome, is also associated with an elevated risk for serious extrahepatic manifestations, including cardiovascular disease, insulin resistance, and type 2 diabetes mellitus (T2DM) (5).

T2DM and NAFLD/NASH share a common pathogenic mechanism, which results in a strong two-way relationship (6). Though the effects of hyperglycemia in patients with T2DM and NAFLD have not been completely elucidated (7), elevated glucose levels and hyperinsulinemia are among the potential biological mechanisms suggested for the progression of liver fibrosis (8). Therefore, the comorbid existence of diabetes in adult NAFLD is an important leading cause of advanced fibrosis and cirrhosis and is further a predictor of liver-related mortality (1, 9).

Compared with adults, the prevalence and effect of T2DM in children with NAFLD have been less well investigated. However, it has also been reported that children with T2DM have a greater risk of developing NASH, which in the long term has significant adverse effects on the liver (10). Hence, identifying the characteristics of pediatric patients with NAFLD with T2DM and evaluating the stage of liver fibrosis are the bases for the prognostic evaluation of NAFLD. However, even in adults, liver biopsies are invasive, inconvenient, and often related to severe morbidity and mortality. Moreover, pediatric patients with NAFLD often have a liver histology that differs from that in adults (11). To overcome these challenges, several non-invasive methods have been used to predict the presence and risk stratification of liver fibrosis in patients with NAFLD. These methods include both non-invasive fibrosis markers using serological tests and radiological imaging techniques using transient elastography (TE, FibroScan®) (12, 13).

TE can determine the degree of hepatic fibrosis using a liver stiffness measurement (LSM) and quantify hepatic steatosis by measuring the ultrasonic attenuation of the echo wave, termed as the controlled attenuation parameter (CAP) (13). A non-invasive fibrosis scoring system was developed by measuring clinical and laboratory parameters to identify advanced fibrosis, including the aspartate aminotransferase (AST)/alanine aminotransferase (ALT) ratio, NAFLD fibrosis score (NFS) (14), AST: platelet ratio index (APRI) (15), Fibrosis-4 (FIB-4) (16), and pediatric NAFLD fibrosis index (PNFI) (17).

The majority of these non-invasive methods have been investigated in adults, but in pediatric patients—due to the lack of data and uncertainties concerning the accuracy of these methods—no consensus has been reached to date (18, 19). Furthermore, their functioning across the entire spectrum of patients with NAFLD, including those of body mass index (BMI), race, and T2DM, has yet to be well established. Therefore, in response to these unmet needs, we conducted this study to investigate the performance of non-invasive diagnostic methods and the association with T2DM in pediatric patients with NAFLD.

## Methods

### Study Design and Patients

We performed this retrospective cross-sectional study by collecting data from electronic medical records between January 2010 and December 2020 with an International Classification of Diseases Code for NAFLD/NASH (K76.0, K75.81) at a tertiary referral hospital in metropolitan Incheon (Inha University Hospital, Korea). In Supplementary Figure 1, we describe the inclusion and exclusion criteria. We excluded three children with a prior diagnosis of type 1 diabetes from the analysis.

We collected the clinical, laboratory, and demographic data from the patients' medical records. The biochemical data we used to perform the analyses were the test results available at the closest time within a maximum of 30 days from the time of liver ultrasound, considering an interval of 30 days between procedures. We calculated BMI and height and weight standard deviation scores (SDS) using the 2017 Korean children and adolescents growth standard (20). We measured serum insulin concentrations by immunoradiometric assay using an INS-IRMA kit (BioSource, Nivelles, Belgium). We measured insulin resistance using homeostasis model assessment-estimated insulin resistance (HOMA-IR), calculating it as follows: HOMA-IR = fasting insulin (μU/ml) × fasting glucose (mg/dl)/22.5. We considered patients to have T2DM if they met at least one of the following three criteria (21): (1) a hemoglobin A1c (HbA1c) level of ≥6.5%, or (2) a fasting serum glucose level of ≥126 mg/dl, or 3) an existing clinical condition of T2DM and met the above (1) or (2) criteria. The Ethics Committee of Inha University Hospital approved the study protocol (2017-12-009-001).

### Diagnosis by Abdomen Ultrasonography

All the ultrasonography examinations were performed by one or two experienced radiologists, and steatosis was graded as follows based on hyperechogenic liver tissue: the increased discrepancy of echo amplitudes between the liver and kidney and the loss of echoes from the walls of the portal system and diaphragm. The definition of NAFLD adopted in this study was based on the presence of steatosis as assessed by the ultrasound steatosis score: absent (grade 0), mild (grade 1), moderate (grade 2), and severe (grade 3) (22).

### TE Measurement

Vibration-controlled TE was performed under at least a 2-h fasting condition on a FibroScan Touch 502 (Echosens®, Paris, France), using examination conditions recommended by the manufacturer (23). All participants underwent TE using an M or XL probe according to body size. Once the LSM and CAP data could be measured successfully 10 times in a row, the early, less precise values were deleted, and the mean of the last 10 valid measurements was retained. The cutoff values for the CAP of FibroScan were defined as follows: (1) S1 (>11%) was >218 dB/m; (2) S2 (>34%) was >258 dB/m; and (3) S3 (>67%) was >283 dB/m (24, 25). The fibrosis result was measured in kilopascals (kPa). The fibrosis score was as follows: F0 (<5.0 kPa) to F1 (5 ≤ kPa <7), no liver scarring or mild liver scarring; F2 (7 ≤ kPa <8.7), moderate liver scarring; F3 (8.7 ≤ kPa <10.3), severe liver scarring; and F4 (kPa ≥ 10.3), advanced liver scarring (cirrhosis) (24).

### Other Non-invasive Markers of Liver Fibrosis

We calculated the APRI as AST (/upper limit of normal)/platelet count (×109/L) ×100 (15). We calculated FIB-4 as age × AST (IU/L)/platelet count (×109/L) × √ALT (IU/L) (16). We calculated the NFS according to the following formula: −1.675 + 0.037 × age (years) + 0.094 × BMI (kg/m^2^) + 1.13 × impaired fasting glycemia/diabetes (yes = 1, no = 0) + 0.99 × AST/ALT ratio – 0.013 × platelet (×109/L) – 0.66 × albumin (g/dl) (14). We calculated PNFI according to the following formula: 1/(1+e–lp) ×10; lp = −6.539 × loge [age (years)] + 0.207 × waist (cm) + 1.957 × loge [triglycerides (mg/dl)] – 0.074.]. We considered a PNFI score of ≥9 as fibrosis (17).

### Other Non-invasive Markers of Liver Fibrosis

We compared two groups using the independent two-sample *t*-test for continuous variables and Pearson's chi-square test or Fisher's exact test for categorical variables. We considered variables with a *p*-value of ≤ 0.10 in the crude analysis as candidates for the multivariable analysis. We conducted a multivariable logistic regression to determine the association between T2DM, FibroScan results, and abdomen sonography adjusted for age, sex, BMI, and waist circumference. We express the results as odds ratio (OR) and 95% confidence interval (CI). We determined the area under the curve (AUC) to evaluate the ability of the non-invasive fibrosis marker and FibroScan to predict the presence of T2DM in fibrosis stage using receiver operating characteristic (ROC) curves. We compared the AUC as the predictive power of several meaningful variables using the Delong method. We evaluated the optimal cutoff for kPa and CAP using Youden's J index for discriminating T2DM. We considered a two-tailed *P*-value of <0.05 to be significant. We performed all statistical analyses using R Statistical Software (version 3.6.3; R Foundation for Statistical Computing, Vienna, Austria).

## Results

### Clinical and Biochemical Characteristics of Study Subjects

We included a total of 152 eligible pediatric patients with NAFLD in this study (for a flow diagram, see Supplementary Figure 1). Of these, 50 patients had T2DM (32.9%)—a higher proportion than that of the age- and sex-matched control group (10.3%, *n* = 111/1077; *P* < 0.001) (Supplementary Figure 2).

In Table 1 we show the demographic and clinical variables of this study. The age range was 5.9–17.9 years (12.9 ± 2.8). A total of 22 patients were aged <10 years. The mean BMI and BMI-SDS of the total cohort were 28.8 ± 4.7 kg/m^2^ and 1.8 ± 0.4, respectively. The mean age of patients with T2DM was higher than that of patients without T2DM (mean, 13.6 vs. 12.6 years, *P* = 0.026). Girls with NAFLD were significantly more likely to have T2DM than boys with NAFLD (*n* = 27/59, 45.8% vs. *n* = 23/93, 24.7%, *P* = 0.007). Mean BMI (kg/m^2^) was significantly higher in patients with T2DM, but BMI-SDS did not differ significantly between the groups. Height (cm), waist circumference, and diastolic blood pressure (BP) were significantly higher in children with NAFLD with T2DM than in those without T2DM. The mean waist-to-height ratio (WHtR) was 0.6 ± 1.0 with no difference between the groups according to the presence or absence of T2DM.

**Table 1:** Major clinical and biochemical characteristics of children/adolescents with NAFLD.

**Factor**	**Total**	**NAFLD with**	**NAFLD with**	*P-*value^*^
		**non-diabetes (*n* = 102)**	**type 2 diabetes (*n* = 50)**	
Age, years	12.9 ± 2.8	12.6 ± 2.9	13.6 ± 2.5	0.026
Male sex	93 (61.2)	70 (68.6)	23 (46.0)	0.007
Height, cm	158.9 ± 14.5	156.5 ± 15.2	163.8 ± 11.5	0.001
Height-SDS	0.6 ± 1.1	0.5 ± 1.2	0.8 ± 1	0.096
BMI, kg/m^2^	28.8 ± 4.7	28.2 ± 4.7	30.1 ± 4.5	0.017
BMI-SDS	1.8 ± 0.4	1.8 ± 0.4	1.8 ± 0.3	0.442
Waist, cm	95.7 ± 12.9	93.3 ± 12.8	100.2 ± 12.1	0.003
WHtR	0.6 ± 0.1	0.6 ± 0.1	0.6 ± 0.1	0.510
Systolic BP, mmHg	120.1 ± 14.2	118.4 ± 14.2	123.1 ± 13.7	0.063
Diastolic BP, mmHg	70.5 ± 10.2	68.7 ± 10.0	73.8 ± 9.7	0.004
ALT, U/L	98.7 ± 91.1	93.3 ± 91.8	109.7 ± 89.7	0.298
AST, U/L	56.8 ± 52.1	52.6 ± 50.1	65.4 ± 55.5	0.158
Serum glucose, mg/dl	132.8 ± 97.6	96.8 ± 26.4	206.1 ± 140.5	<0.001
HbA1c, %	6.7 ± 2	5.6 ± 0.5	9 ± 2.1	<0.001
Serum insulin, μIU/ml	40.4 ± 68.4	37.8 ± 53.9	45 ± 88.4	0.599
HOMA-IR	12.8 ± 23.2	9 ± 12.5	19.3 ± 33.9	0.044
HDL, mg/dl	43.9 ± 11.1	45.3 ± 11.8	40.9 ± 9	0.012
LDL, mg/dl	115.5 ± 33.5	114.7 ± 30.3	117.1 ± 39.5	0.711
Total cholesterol, mg/dl	176.3 ± 39.1	173.6 ± 31.5	181.9 ± 51.2	0.296
Triglycerides, mg/dl	143.5 ± 71.5	130.6 ± 66.6	169.2 ± 74.6	0.002
Platelet count, 109/L	314.6 ± 89.2	327.1 ± 83.7	289.7 ± 95.3	0.015
Albumin, g/dl	4.7 ± 0.3	4.7 ± 0.3	4.7 ± 0.4	0.298
Uric acid, mg/dl	6.5 ± 1.5	6.6 ± 1.5	6.2 ± 1.4	0.115

*Data are expressed as n (%) or mean ± SD*.

*ALT, alanine aminotransferase; AST, aspartate aminotransferase; BMI, body mass index; WHtR, waist-to-height ratio; BP, blood pressure; HbA1c, hemoglobin A1c; HDL, high-density lipoprotein; HOMA-IR, homeostasis model assessment-estimated insulin resistance; LDL, low-density lipoprotein; NAFLD, non-alcoholic fatty liver disease; SD, standard deviation; SDS, standard deviation scores;*.

* *Significant association was classified as P <0.05*.

Regarding laboratory findings, the mean ALT and AST of the total cohort were 109.7 ± 89.7 and 65.4 ± 55.5 U/L, respectively. The mean serum glucose and HbA1c levels of the NAFLD with T2DM group were 206.1 ± 140.5 mg/dl and 9.0 ± 2.1%, respectively, which were markedly higher than those of the group without T2DM. HOMA-IR and mean serum triglyceride levels were significantly higher in children with T2DM, whereas high-density lipoprotein (HDL) level and platelet count were significantly lower in children with T2DM than those in children without T2DM. Serum insulin, low-density lipoprotein (LDL), total cholesterol, albumin, and uric acid levels did not differ between the two groups. In particular, there was no significant difference in ALT and AST levels of the NAFLD with T2DM group and those of the NAFLD without T2DM group.

### Comparison of Non-invasive Fibrosis Markers and Imaging Assessment

We analyzed all subjects by comparing the non-invasive fibrosis markers (NFS, APRI, PNFI, and FIB-4) of the NAFLD with T2DM group with those of the NAFLD without T2DM group (Table 2). Risk stratification was as follows: NFS [low probability of fibrosis (< -1.455), indeterminate (−1.455 to −0.675), high probability of fibrosis (>0.675)], APRI [no (<0.5), indeterminate (0.5–1.5), advanced fibrosis (≥1.5), cirrhosis (>2)], PNFI (non-fibrosis, fibrosis), and FIB-4 [no (<1.45)/indeterminate (1.45–3.25)/advanced fibrosis (>3.25)]. Patients with T2DM and NAFLD had a significantly higher mean NFS score (−1.2 vs. −2.9, *P* < 0.001) than that of nondiabetic patients with NAFLD, and we also observed a significant difference between the two groups in risk stratification (*P* < 0.001). However, we found no significant differences in the mean score of APRI and PNFI between the two groups. FIB-4 was significantly higher in patients with T2DM, but there was no significant distribution between the two groups when comparing risk-stratified FIB-4.

**Table 2:** Non-invasive fibrosis markers and non-invasive imaging assessment in children/adolescents with NAFLD.

**Non-invasive methods**	**Total**	**NAFLD with non-diabetes**	**NAFLD with type 2 diabetes**	*P-*value^*^
NFS	−2.3 ± 1.9	−2.9 ± 1.7	−1.2 ± 1.8	<0.001
Risk stratification by NFS				<0.001
Low probability of fibrosis	100 (67.1)	82 (82.8)	18 (36.0)	
Indeterminate	46 (30.9)	17 (17.2)	29 (58.0)	
High probability of fibrosis	3 (2.0)	0 (0.0)	3 (6.0)	
APRI	0.5 ± 0.5	0.4 ± 0.4	0.6 ± 0.6	0.053
Risk stratification by APRI				0.082
No (<0.5)	101 (67.3)	72 (72.0)	29 (58.0)	
Indeterminate (0.5–1.5)	39 (26)	24 (24.0)	15 (30.0)	
Advanced fibrosis (1.5–2)	5 (3.3)	3 (3.0)	2 (4.0)	
Cirrhosis (>2)	5 (3.3)	1 (1.0)	4 (8.0)	
PNFI	8.3 ± 2.3	8.0 ± 2.4	8.8 ± 2.0	0.055
Risk stratification by PNFI				0.041
Non-fibrosis	52 (40.9)	39 (47.6)	13 (28.9)	
Fibrosis	75 (59.1)	43 (52.4)	32 (71.1)	
FIB-4	0.3 ± 0.1	0.2 ± 0.1	0.3 ± 0.2	0.009
Risk stratification by FIB-4				>0.999
No (<1.45)	150 (100)	100 (100)	50 (100)	
Indeterminate (1.45–3.25)	0 (0.0)	0 (0.0)	0 (0.0)	
Advanced fibrosis (>3.25)	0 (0.0)	0 (0.0)	0 (0.0)	
Abdomen sonography				0.002
Mild	51 (33.6)	43 (42.2)	8 (16)	
Moderate	89 (58.6)	54 (52.9)	35 (70)	
Severe	12 (7.9)	5 (4.9)	7 (14)	
FibroScan				
LSM by TE, kPa	7.1 (2.7)	6.8 (2.6)	7.4 (2.8)	0.397
CAP, dB/m	328.2 (42.8)	312.1 (41.6)	343.7 (38.6)	0.004
Fibrosis grade				0.49
F0, Normal	15 (25.4)	8 (26.7)	7 (24.1)	
F1, Low-grade insignificant	20 (33.9)	13 (43.3)	7 (24.1)	
F2, Low-grade fibrosis	14 (23.7)	5 (16.7)	9 (31)	
F3, Advanced fibrosis	3 (5.1)	1 (3.3)	2 (6.9)	
F4, Cirrhosis	7 (11.9)	3 (10)	4 (13.8)	
Steatosis grade				0.214
S0, Normal	1 (1.8)	1 (3.6)	0 (0)	
S1, >11%	0 (0.0)	0 (0.0)	0 (0.0)	
S2, >34%	9 (15.8)	6 (21.4)	3 (10.3)	
S3, >67%	47 (82.5)	21 (75)	26 (89.7)	

*Data are expressed as n (%) or mean ± SD*.

*APRI, AST: platelet ratio index; CAP, controlled attenuation parameter; FIB-4, Fibrosis-4 index; LSM, liver stiffness measurement; NAFLD, non-alcoholic fatty liver disease; NFS, NAFLD fibrosis score; PNFI, pediatric NAFLD fibrosis index; SD, standard deviation; TE, transient elastography*.

* *Significant association was classified as P <0.05*.

We also performed a comparison analysis of the non-invasive imaging results of patients based on the presence of T2DM (Table 2). First, we classified the results of abdominal ultrasonography into mild, moderate, and severe types, and observed significant differences between the two groups (*P* = 0.002). Especially, the moderate-to-severe types were more common in the T2DM group (84 vs. 57.8%, respectively). FibroScan was performed in 59 patients, and the mean LSM value of the T2DM group was 7.4 ± 2.8 kPa, which was higher than that (6.8 ± 2.6 kPa) of the nondiabetic group (*P* = 0.397), but not significant. The CAP value was significantly higher in the T2DM group (343.7 ± 38.6 dB/m) than that in the nondiabetic group (312.1 ± 41.6; *P* = 0.004). Though the two groups showed no differences according to the fibrosis grade and steatosis grade, F2–F4 accounted for a considerably larger proportion in patients with T2DM, and S3 was also relatively higher than that in patients without T2DM.

### Impact of Non-invasive Fibrosis Markers and Non-invasive Imaging on T2DM

#### ROC Curves of Non-invasive Fibrosis Markers and Non-invasive Imaging Results

**Figure 1:**
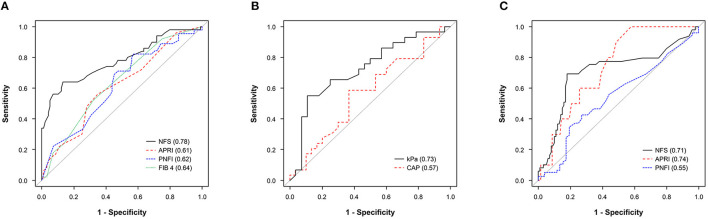
**(A)** ROC curve of NFS, APRI, PNFI, and FIB-4 in predicting the presence of T2DM. **(B)** ROC curve of kPa and CAP in predicting the presence of T2DM. **(C)** ROC curve of HbA1c (%) in predicting any fibrosis progression from NFS (intermediate to high probability fibrosis vs. low probability of fibrosis), APRI (advanced fibrosis to cirrhosis vs. no to indeterminate), and PNFI (fibrosis vs. non-fibrosis). APRI, AST: platelet ratio index; CAP, controlled attenuation parameter; FIB-4, Fibrosis-4 index; HbA1c, hemoglobin A1c; NAFLD, non-alcoholic fatty liver disease; NFS, NAFLD fibrosis score; OR, odds ratio; PNFI, pediatric NAFLD fibrosis index; ROC, receiver operating characteristic; T2DM, type 2 diabetes mellitus.

In Figure 1A we show the ROC curves of the non-invasive fibrosis markers with NFS, APRI, PNFI, and FIB-4 for the presence of T2DM in pediatric patients with NAFLD. Among them, NFS showed the best performance (AUC, 0.78; 95% CI, 0.69–0.87) for detecting the presence of T2DM, followed by FIB-4 (AUC, 0.64; 95% CI, 0.55–0.73), PNFI (AUC, 0.62; 95% CI, 0.52–0.72), and APRI (AUC, 0.61; 95% CI, 0.52–0.71). NFS had a sensitivity of 64% and a specificity of 87% (maximum Youden's J index) with a cutoff value of −1.35, which showed a significant difference compared with those of FIB-4 (*P* = 0.005), PNFI (*P* = 0.004), and APRI (*P* = 0.004). The cutoff values for detecting the presence of T2DM were 0.25 for FIB-4, 8.55 for PNFI, and 0.35 for APRI.

The AUROC (95% CI) of kPa and CAP for detecting the presence of T2DM were 0.57 (95% CI, 0.42–0.72) and 0.73 (95% CI, 0.59–0.86), respectively (Figure 1B). The cutoff values were 6.9 for kPa (OR = 2.45, *P* = 0.094) and 348.5 for CAP (OR = 10.26, *P* = 0.001). The AUROCs of HbA1c (%) in predicting any fibrosis progression using the non-invasive fibrosis markers (Figure 1C) were 0.74 (95% CI, 0.62–0.87) for APRI predicting from advanced fibrosis to cirrhosis, 0.71 (95% CI, 0.61–0.81) for NFS predicting from intermediate score to high probability fibrosis, and 0.55 for PNFI predicting fibrosis (≥9 points) (95% CI, 0.44–0.65), respectively. The optimal HbA1c cutoff value for predicting any fibrosis progression in pediatric patients with NAFLD in APRI, NFS, and PNFI were 5.7, 6.4, and 6.4%, respectively. In Supplementary Tables 1–3 we show the predictive performance of each ROC curve and the sensitivity, specificity, positive predictive value, negative predictive value, and accuracy values.

#### Multivariable Regression Analysis for Predictors of T2DM

In Table 3 we show crude and adjusted ORs for the logistic regression model with presence of T2DM as a dependent variable and non-invasive methods as independent variables. In the unadjusted model, we found moderate-to-severe hepatosteatosis on abdomen sonography, NFS with intermediate score to high probability of fibrosis, and PNFI value predicting fibrosis to be significantly associated with the presence of T2DM (*P* < 0.05). In the multivariable analysis, hepatosteatosis on abdomen sonography (OR, 5.80; 95% CI, 1.85–18.16; *P* = 0.003) and NFS (OR, 9.91; 95% CI, 3.64–26.95; *P* < 0.001) were independently associated with T2DM risk. The FibroScan F result value of ≥F2 and APRI value predicting advanced fibrosis to cirrhosis showed a slight significant association with the presence of T2DM in the crude model (*P* < 0.1), but the adjusted analysis revealed an independently significant OR [(OR, 7.25; 95% CI, 1.51–34.90; *P* = 0.013), (OR, 9.47; 95% CI, 1.40–64.17; *P* = 0.021), respectively].

**Table 3:** Crude and adjusted odds ratios based on a logistic regression analysis with type 2 diabetes as a dependent variable.

**Variables**	**Crude**		**Adjusted^a^**	
	**OR (95% CI)**	*P*-value^*^	**OR (95% CI)**	*P*-value^*^
Fibroscan S (≥S2)	N/A		N/A	
Fibroscan F (≥F2)	2.50 (0.86–7.27)	0.093	7.25 (1.51–34.90)	0.013
Abdomen sonography (moderate-to-severe hepatic steatosis)	3.83 (1.63–8.97)	0.002	5.80 (1.85–18.16)	0.003
NFS (intermediate score to high probability fibrosis)	8.57 (3.94–18.68)	<0.001	9.91 (3.64–26.95)	<0.001
APRI (advanced fibrosis to cirrhosis)	3.27 (0.88–12.18)	0.077	9.47 (1.4–64.17)	0.021
PNFI (fibrosis)	2.23 (1.03–4.86)	0.043	2.81 (0.85–9.31)	0.092
FIB-4	N/A		N/A	

*APRI, AST: platelet ratio index; CI, confidence interval; FIB-4, Fibrosis-4 index; NAFLD, non-alcoholic fatty liver disease; NFS, NAFLD fibrosis score; OR, odds ratio; PNFI, pediatric NAFLD fibrosis index; N/A, not applicable*.

* *Significant association was classified as P <0.05*.

a *Multivariable model adjusted for age, sex, BMI, and waist circumference*.

PNFI did not differ significantly according to severity, and we could not analyze FIB-4 values according to severity, as there was no value for indeterminate or advanced fibrosis. We dropped Fibroscan S (≥S2) from logistic regression because of a zero cell (failure of model to converge) (26). Liver biopsy was performed in 11 patients, of whom seven were histologically diagnosed with NASH (Supplementary Table 4).

## Discussion

This study examines the relational implications of noninvasive fibrosis markers and imaging methods in pediatric NAFLD as follows: (i) the reliability of NFS as a noninvasive method to predict the coexistence risk of T2DM; (ii) the relationship between the performance of noninvasive fibrosis markers and the HbA1c cutoff value as a fibrosis predictive model; (iii) close association of noninvasive fibrosis markers and TE with T2DM through multivariate regression analysis.

The clinical characteristics of our patients with T2DM were similar to those of KP Newton et al. (10) in that age, height, weight, BMI, BP, waist circumference, the proportion of females, and triglycerides were higher than those in the nondiabetic group. Notably, in both studies, the ALT level in the T2DM-NAFLD group was not significantly higher than that in the nondiabetic group. The increase in ALT is known to be closely related to the presence of T2DM (27), but it may lead to such a result because the ALT is not necessarily elevated in NAFLD patients (28). We also found that lower platelet counts, higher HOMA-IR, and lower HDL levels were found in NAFLD patients with T2DM. The mean value of WHtR was 0.6 ± 0.1, which was the same in the diabetic and nondiabetic groups, but was higher than 0.5, the cutoff value associated with increased cardiometabolic risk in adults and children (29, 30). The abnormal cardiometabolic profile (high BP, high HOMA-IR, high WHtR and dyslipidemic profile) found in this study is in line with that of previous studies revealing the cardiometabolic risk burden associated with NAFLD in children (31–35).

In this study, the prevalence of T2DM in children with NAFLD was 32.9%—higher than that of previous studies (10, 36). This may be related to the increased proportion of obese patients in the process of including only NAFLD pediatric patients who underwent HbA1c testing to facilitate a comparative study. Moreover, in a previous study that followed patients with NAFLD for more than 4 years, the risk of developing T2DM was 1.3 to 5-fold higher than that in patients without NAFLD (37, 38). Given these findings—and the characteristics of our patient cohort—the relatively high prevalence of T2DM in our study may be explained.

FibroScan and the non-invasive fibrosis markers used in this study to evaluate the degree of liver fibrosis have lately emerged as methods to replace invasive liver biopsy in adults. A recent study showed that non-invasive methods (FIB-4, APRI, and FibroScan) provide results that are similar to those obtained using liver biopsy (39). FibroScan reportedly has high reliability as a result of comparing META analysis of histological data with VIRal hepatitis (METAVIR) score with liver biopsy (40, 41). Non-invasive fibrosis markers such as NFS, APRI, and FIB-4 are also demonstrably reliable methods in NAFLD in adults (42). The PNFI, the only fibrosis score developed for pediatric patients, remains controversial regarding the accuracy and usefulness of the results (18). However, a recent study showed that the combination of PNFI and TE in pediatric NAFLD could be a useful marker in predicting clinically significant fibrosis, although more research evidence is needed (43).

In this study, NFS and FIB-4 were significantly higher in pediatric patients with NAFLD with T2DM than in those without T2DM, but with a lesser degree of risk stratification than that observed in adults. This may be due to the progression of fibrosis in children is not as advanced as that in adults, and its absolute value is lower. In addition, the TE result of T2DM-NAFLD patients was quite high, with a cutoff of 7.4 kPa and 343.7 CAP, which suggest the possibility of liver fibrosis progression in the coexistence of T2DM in pediatric NAFLD patients. In light of these results, evaluating fibrosis using non-invasive fibrosis markers alone in pediatric patients runs the risk of missing fibrosis; hence, it is recommended that imaging assessments such as TE be performed together as in the aforementioned Alkhouri et al. (43). Lomonaco et al. (44) reported that the combined use of non-fibrosis markers and TE improves diagnostic performance when diagnosing NAFLD or NASH in patients with T2DM. The American Diabetes Association guideline for adult patients with NAFLD and T2DM (45) also suggested the assessment for of fibrosis risk by performing TE and non-invasive biomarker tests for pediatric patients with NAFLD with T2DM—a recommendation supported by our study. Another interesting observation is that the HbA1c cutoff values of NFS and APRI—which are reliable in predicting progression to severe fibrosis in pediatric patients with T2DM—were 6.4 and 5.7%, respectively (Figure 1C), which suggests the possibility of fibrosis progression in the prediabetes stage. In other words, there exists a need to actively monitor and predict the progression of severe fibrosis using non-invasive methods (imaging and markers) in the prediabetes stage in pediatric patients with NAFLD.

Our findings demonstrated that the TE cutoff value for predicting T2DM and NAFLD comorbidities was kPa ≥ 6.9 (>F1) and CAP ≥ 348.5 (>S3). Although the kPa cutoff value was not relatively high, the comorbid condition with T2DM and NAFLD should be kept under surveillance because it may act as a “rapid progressor” in the early stage of F1 and has a high possibility of progressing to severe fibrosis (44). Furthermore, considering that the majority of patients with moderate-to-advanced fibrosis (F2 or higher) have severe steatosis (S3), the steatosis of pediatric patients with NAFLD with T2DM is severe, suggesting that the probability of progression to more-than-moderate fibrosis is high. In Table 3 we also illustrate that children with NAFLD are more likely to develop T2DM when the degree of fibrosis or steatosis is severe—results that can be further explained by the prevalence rate of diabetes of 8.5% in the general population, compared with the prevalence rates of diabetes in patients with NAFLD and NASH of 22.51 and 43.61%, respectively, which are significantly higher (46).

The NFS demonstrated an overall good performance not only as a model for predicting the coexistence of T2DM (Figure 1A; Table 3) but also as a model for predicting the possibility of progressing to a severe fibrosis stage (Figure 1C). The NFS has proved to be a marker of advanced fibrosis in patients with NAFLD, while also being linked to cardiovascular disease risk factors in diverse populations with or without NAFLD. In addition, HbA1c is reportedly an independent determinant of NFS in patients with T2DM (47). Given these results, in pediatric patients with impaired glycemic control and NAFLD, the NFS is recommended as a non-fibrosis marker that should be checked first.

Our study had several limitations. First, because of the retrospective design conducted with a small number of samples, the present study has limited ability to elucidate the exact causal relationship between non-invasive methods and T2DM in pediatric patients with NAFLD. Second, liver biopsy, the standard measurement of fat infiltration in the liver, was not conducted on all patients. However, for use in the clinical setting, ultrasonography is a sensitive and reasonably non-invasive surrogate method. Third, because we used HbA1c level to diagnose T2DM in addition to medical history, without subjecting the patients to oral glucose tolerance testing, the prevalence may be underestimated or overestimated. Finally, this is a single center study, which has the potential for selection bias. Future studies should therefore implement a validation design across different populations and multiple centers. Despite these limitations, our study generated several remarkable results. Most notably, to our knowledge, this is the first study using FibroScan and non-invasive fibrosis markers in pediatric patients with NAFLD with diabetic comorbidity. We believe future major research design for confirmation should incorporate large-scale studies to derive meaningful results. Despite the retrospective design of our study, our results are significant in that they compared matching groups with similar BMI and age. Studies on children and adolescents are extremely rare, and there are currently no guidelines for assessing NAFLD and T2DM in these groups. The unique value of this study lies in its use as a reference in the steps for establishing guidelines and developing and applying future treatments.

## Conclusion

The NFS is a reliable non-invasive method/ for predicting T2DM comorbidities and fibrosis progression in children and adolescents with NAFLD. The combination of a non-invasive fibrosis marker and TE provide a tool for evaluating the risk of developing T2DM, which itself poses a high risk of fibrosis progression in pediatric patients with NAFLD. Considering the fairly high prevalence of T2DM among pediatric patients with NAFLD, the identification of T2DM comorbidity should be comprehensively performed. We also strongly advocate the evaluation of analysis fibrosis progression from the prediabetes stage using non-invasive methods.

## Data Availability Statement

The original contributions presented in the study are included in the article/Supplementary Material, further inquiries can be directed to the corresponding author/s.

## Ethics Statement

The study with human subjects was reviewed and approved by the Inha University Hospital Ethics Committee (2017-12-009-001). Written informed consent from the participants' legal guardian/next of kin was not required to participate in this study in accordance with the national legislation and the institutional requirements.

## Author Contributions

NJ collected the data and drafted the manuscript. SK performed statistical analyses and data interpretation. J-EL contributed to study design and takes responsibility for the integrity of data as well as for the accuracy of data analysis. AY conceived the idea, supervised the study, interpreted results, and drafted the manuscript. All authors approved the final version and agree to be accountable for all aspects of the work.

## Funding

This research was supported by the SungKyunKwan University and the BK21 FOUR (Graduate School Innovation) funded by the Ministry of Education (MOE, Korea) and National Research Foundation of Korea (NRF).

## Conflict of Interest

The authors declare that the research was conducted in the absence of any commercial or financial relationships that could be construed as a potential conflict of interest.

## Publisher's Note

All claims expressed in this article are solely those of the authors and do not necessarily represent those of their affiliated organizations, or those of the publisher, the editors and the reviewers. Any product that may be evaluated in this article, or claim that may be made by its manufacturer, is not guaranteed or endorsed by the publisher.
