# Supplementary material for: Association Between Non-invasive Diagnostic Methods of Liver Fibrosis and Type 2 Diabetes in Pediatric Patients With Non-alcoholic Fatty Liver Disease
Source: Front Pediatr. 2022 Feb 10;10:825141. doi: 10.3389/fped.2022.825141 (PMC8866638; doi:10.3389/fped.2022.825141)
Supplement: Supplementary file 1 [file Data_Sheet_1.docx]

Supplementary Material

# Supplementary Tables

| **Supplementary Table 1.** Prediction performance of non-invasive fibrosis models to detect presence of T2DM. | | | | | | | |
| --- | --- | --- | --- | --- | --- | --- | --- |
|  | **AUC (95% CI)** | **Optimal cut off point** | **Sensitivity (95% CI)** | **Specificity (95% CI)** | **PPV (95% CI)** | **NPV (95% CI)** | **Accuracy (95% CI)** |
| FIB-4 | 0.637 (0.550-0.725)^**^ | ≥0.25 | 0.520 (0.382-0.658) | 0.680 (0.589-0.771) | 0.448 (0.320-0.576) | 0.739 (0.649-0.829) | 0.627 (0.624-0.630) |
| APRI | 0.613 (0.519-0.707)^**^ | ≥0.35 | 0.560 (0.422-0.698) | 0.650 (0.557-0.743) | 0.444 (0.322-0.567) | 0.747 (0.656-0.838) | 0.620 (0.617-0.623) |
| NFS | 0.779 (0.694-0.865)^**^ | ≥-1.35 | 0.640 (0.507-0.773) | 0.869 (0.802-0.935) | 0.711 (0.579-0.844) | 0.827 (0.754-0.900) | 0.792 (0.790-0.794) |
| PNFI | 0.621 (0.519-0.722)^**^ | ≥8.55 | 0.822 (0.711-0.934) | 0.427 (0.320-0.534) | 0.440 (0.334-0.547) | 0.814 (0.698-0.930) | 0.567 (0.563-0.571) |
| APRI, AST:platelet ratio index; AUC, area under the curve; CI, confidence interval; FIB-4, Fibrosis-4 index; NFS, NAFLD fibrosis score; NPV, negative predictive value; PNFI, pediatric NAFLD fibrosis index; PPV, positive predictive value; T2DM, type 2 diabetes mellitus.  ^**^*P*<0.01 compared with NFS vs FIB-4 or APRI or PNFI using Delong’s method. | | | | | | | |

| **Supplementary Table 2.** Prediction performance of kPa and CAP to detect presence of T2DM. | | | | | | | |
| --- | --- | --- | --- | --- | --- | --- | --- |
|  | **AUC (95% CI)** | **Optimal cut off point** | **Sensitivity (95% CI)** | **Specificity (95% CI)** | **PPV (95% CI)** | **NPV(95% CI)** | **Accuracy (95% CI)** |
| kPa | 0.568 (0.419-0.717) | ≥6.9 | 0.586 (0.407-0.765) | 0.633 (0.461-0.806) | 0.607 (0.426-0.788) | 0.613 (0.441-0.784) | 0.610 (0.602-0.618) |
| CAP | 0.727 (0.592-0.862) | ≥348.5 | 0.552 (0.371-0.733) | 0.893 (0.778-1.007) | 0.842 (0.678-1.006) | 0.658 (0.507-0.809) | 0.719 (0.712-0.726) |
| AUC, area under the curve; CAP, controlled attenuation parameter; CI, confidence interval; NPV, negative predictive value; PPV, positive predictive value; T2DM, type 2 diabetes mellitus. | | | | | | | |

| **Supplementary Table 3.** Prediction performance of any fibrosis progression from non-invasive fibrosis scores. | | | | | | | |
| --- | --- | --- | --- | --- | --- | --- | --- |
|  | **AUC (95% CI)** | **Optimal cut off point** | **Sensitivity (95% CI)** | **Specificity (95% CI)** | **PPV (95% CI)** | **NPV (95% CI)** | **Accuracy (95% CI)** |
| FIB-4 | N/A | - |  |  |  |  |  |
| APRI | 0.741 (0.617-0.865) | ≥5.65 | 1.000 (1.000-1.000) | 0.421 (0.340-0.503) | 0.110 (0.046-0.174) | 1.000 (1.000-1.000) | 0.460 (0.457-0.463) |
| NFS | 0.710 (0.611-0.808) | ≥6.35 | 0.694 (0.565-0.823) | 0.820 (0.745-0.895) | 0.654 (0.525-0.783) | 0.845 (0.773-0.917) | 0.779 (0.776-0.781) |
| PNFI | 0.546 (0.443-0.648) | ≥6.35 | 0.427 (0.315-0.539) | 0.731 (0.610-0.851) | 0.696 (0.563-0.829) | 0.469 (0.360-0.578) | 0.551 (0.547-0.555) |
| APRI, AST:platelet ratio index; AUC, area under the curve; CI, confidence interval; FIB-4, Fibrosis-4 index; NFS, NAFLD fibrosis score; NPV, negative predictive value; PNFI, pediatric NAFLD fibrosis index; PPV, positive predictive value. | | | | | | | |

| **Supplementary Table 4.** Liver histology in our patients diagnosed with NASH. | |
| --- | --- |
| **Liver histology** | **NASH (n = 7)** |
| Steatosis grade, % |  |
| Gr1 (<33) | 4 |
| Gr2 (34-66) | 1 |
| Gr3 (>66) | 2 |
| Fibrosis stage |  |
| 1: Mild, zone 3 perisinusoidal | 6 |
| 2: Zone3 and periportal | 1 |
| 3: Bridging |  |
| 4: Cirrhosis |  |
| NASH, nonalcoholic steatohepatitis. | |

# Supplementary Figures


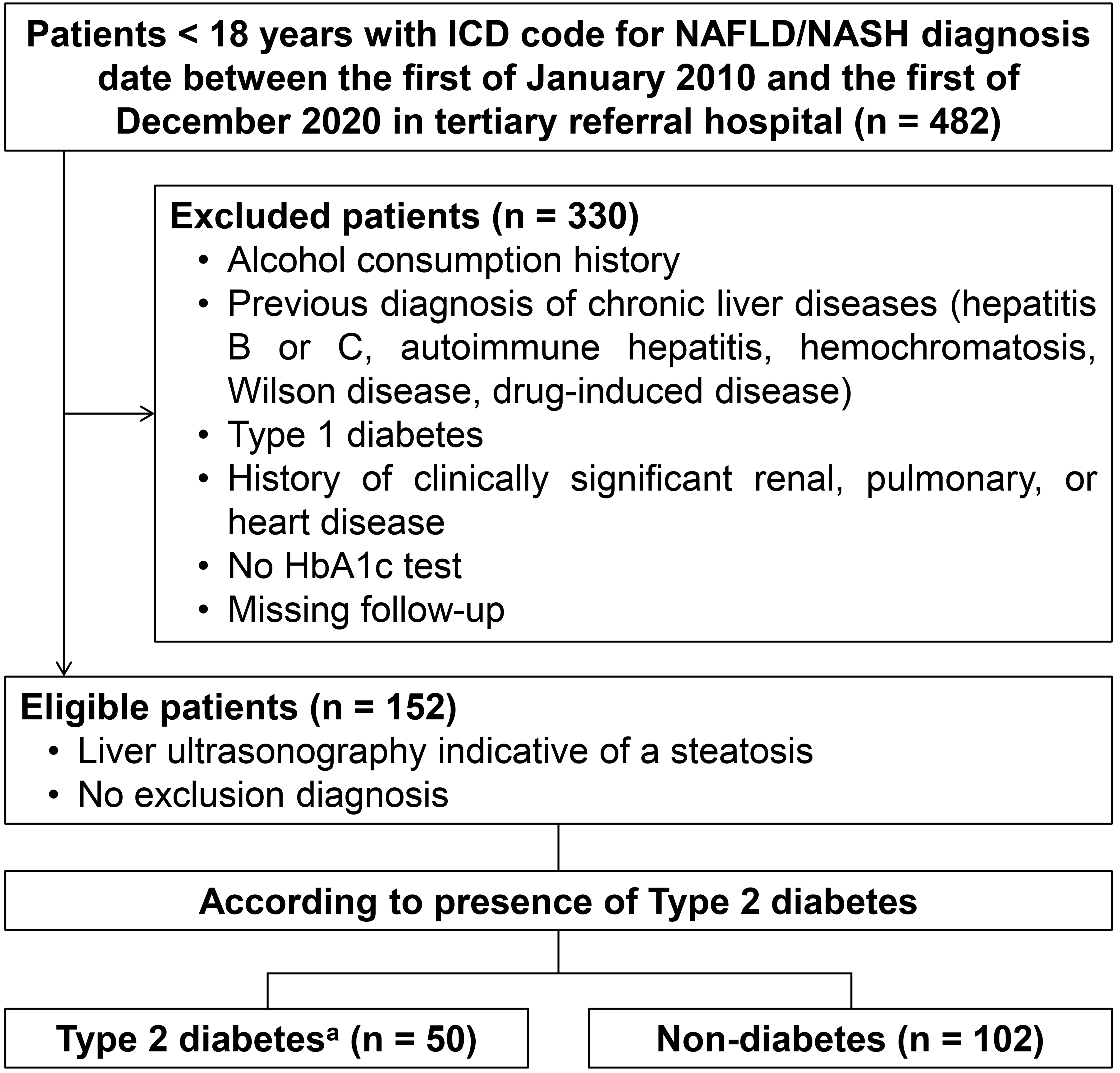


**Supplementary Figure 1.** Selection and entry of study subjects in this study.

Diagnosis of type 1 diabetes was made by synthesizing findings such as clinical features, autoantibody positivity, and reduction of serum C-peptide while satisfying the ADA diagnostic criteria for diabetes through chart review.


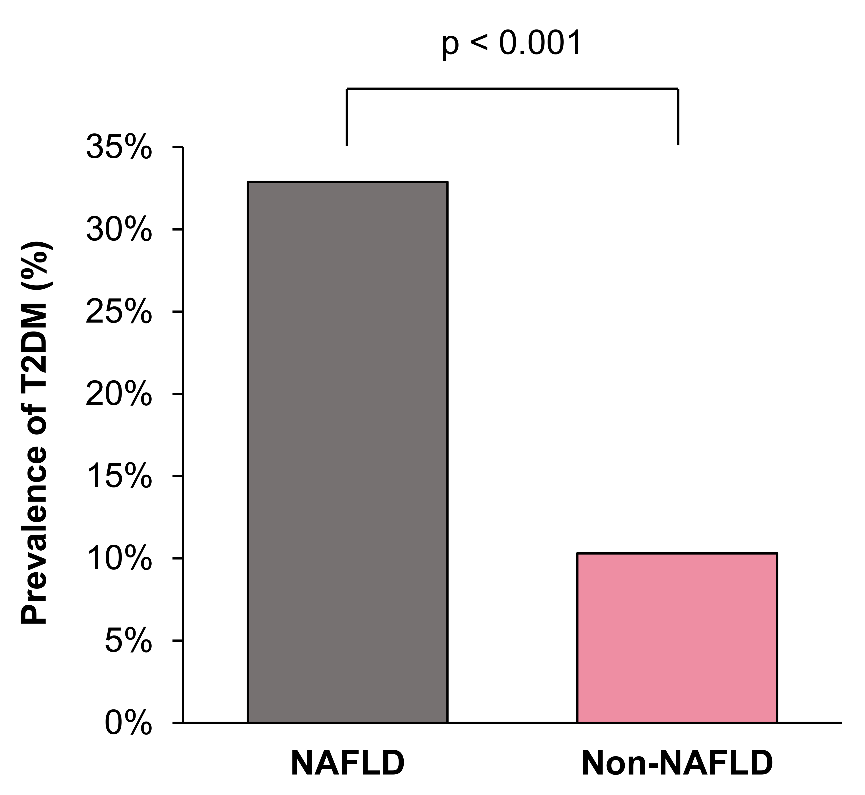


**Supplementary Figure 2.** Proportion of children/adolescents with type 2 diabetes according to the presence of NAFLD status at the time of this study.
NAFLD, non-alcoholic fatty liver disease.
